# Acquired immune-mediated thrombotic thrombocytopenic purpura with severe cholestatic liver injury and reversible acute kidney injury after ivonescimab-containing chemoimmunotherapy: a case report and literature review

**DOI:** 10.3389/fimmu.2026.1908239

**Published:** 2026-08-04

**Authors:** Qiqi Chen, Xiaojing Song, Ming Zhang, Hongxia Niu, Wei Guo

**Affiliations:** 1 Department of Emergency, Beijing Electric Power Hospital of State Grid Corporation of China, Beijing, China; 2 Department of Emergency, Peking University People’s Hospital, Beijing, China; 3 Department of Cardiology, Beijing Electric Power Hospital of State Grid Corporation of China, Beijing, China

**Keywords:** acute kidney injury, ADAMTS13, cholestatic liver injury, immune-mediated thrombotic thrombocytopenic purpura, immune-related adverse event - irAE, ivonescimab, PD-1/VEGF bispecific antibody, thrombotic microangiopathy

## Abstract

**Background:**

Ivonescimab is a PD-1/VEGF bispecific antibody that combines immune checkpoint blockade with anti-angiogenic activity. As PD-1/VEGF-directed regimens enter routine solid-tumor care, rare toxicities at the interface of immune activation, endothelial injury, and thrombotic microangiopathy require careful case-level characterization.

**Case presentation:**

A 63-year-old man with stage IVB lung adenocarcinoma (cT1bN2M1c, with multiple extrathoracic rib metastases) received an ivonescimab-containing combination regimen consisting of three cycles of ivonescimab plus nab-paclitaxel and cisplatin, followed by chemotherapy alone after partial response. He developed fever, severe cholestatic liver injury, and KDIGO stage 3 acute kidney injury requiring temporary continuous renal replacement therapy. Serum creatinine peaked at 767 µmol/L and subsequently normalized. Evolving anemia, thrombocytopenia, schistocytes, Coombs-negative hemolysis, and organ injury prompted evaluation for thrombotic microangiopathy. ADAMTS13 activity was 8.87% with a functional inhibitor of 1.8 BU/mL, confirming acquired immune-mediated thrombotic thrombocytopenic purpura. The PLASMIC score was 5, reflecting intermediate clinical probability because active malignancy and MCV >90 fL lowered the score; definitive diagnosis therefore depended on ADAMTS13 testing. Treatment included corticosteroids, 10 sessions of DPMAS/TPE support with transition to FFP-based plasma exchange once iTTP was recognized, and rituximab 500 mg weekly from April 17 to May 8, 2026. Caplacizumab was not used because it was unavailable locally. No platelet transfusions were administered; red blood cells and plasma were transfused as clinically indicated. ADAMTS13 activity increased to 23.82% and inhibitor titer decreased to 0.85 BU/mL on the available follow-up test, but serial ADAMTS13 and complement measurements were not available. The patient had biochemical improvement and normalization of renal function.

**Conclusion:**

This case highlights ADAMTS13 inhibitor-positive iTTP as a rare, actionable toxicity after ivonescimab-containing chemoimmunotherapy rather than proof of ivonescimab as the sole causal agent. The renal phenotype should be framed as severe but reversible AKI rather than dialysis-dependent renal failure. In patients receiving PD-1/VEGF-directed combination therapy, thrombocytopenia with microangiopathic hemolysis and organ injury should trigger smear review, PLASMIC scoring, urgent ADAMTS13 testing, and early mechanism-directed therapy.

## 1 Introduction

Immune checkpoint inhibitors have reshaped the management of advanced solid tumors, but their clinical benefit is accompanied by immune-related adverse events that may involve virtually any organ system (1, 2). Ivonescimab is a tetravalent PD-1/VEGF bispecific antibody designed to integrate checkpoint blockade and anti-angiogenic activity in a single molecule. Randomized phase 3 studies in non-small cell lung cancer have shown clinically meaningful activity of ivonescimab-containing regimens (3, 4).

Acquired immune-mediated thrombotic thrombocytopenic purpura (iTTP) is a rare but life-threatening thrombotic microangiopathy caused by severe ADAMTS13 deficiency, usually mediated by inhibitory autoantibodies (5). Clinical prediction tools such as the PLASMIC score can help triage suspected thrombotic microangiopathy before ADAMTS13 results return, but ADAMTS13 activity and inhibitor testing remain decisive when the presentation is confounded by malignancy, infection, chemotherapy, renal injury, or liver dysfunction (5, 6). International consensus definitions distinguish laboratory improvement from formal clinical response, clinical remission, exacerbation, relapse, and ADAMTS13 remission (7). Current iTTP management guidance supports urgent therapeutic plasma exchange and immunosuppression (8, 9). Caplacizumab and rituximab may be considered according to drug availability, bleeding risk, relapse risk, and clinical context (10, 11).

Pharmacovigilance analyses and early case reports support a rare ICI-associated TTP signal (12–14). Cases after nivolumab, checkpoint inhibitor immunotherapy, and metastatic non-small-cell lung cancer treatment illustrate the diagnostic heterogeneity and variable severity of this toxicity (15–17). Pembrolizumab-associated reports and dual-checkpoint cases further show that outcomes range from fatal disease to durable remission with extended plasma exchange, rituximab, and ADAMTS13 monitoring (18–20). More recent nivolumab-, pembrolizumab-, and PD-L1/taxane-associated cases extend the phenotype across checkpoint classes and combination regimens (21–23). Anti-VEGF therapy can independently promote glomerular endothelial injury and renal thrombotic microangiopathy (24, 25). Rather than proposing a new toxicity entity, the present case documents confirmed ADAMTS13 inhibitor-positive iTTP in a PD-1/VEGF bispecific antibody-containing chemoimmunotherapy setting, where immune activation, endothelial injury, cholestatic liver injury, and reversible AKI overlapped.

## 2 Case presentation

### 2.1 Oncologic background and treatment exposure

A 63-year-old man had coronary artery disease and benign prostatic hyperplasia, with no known autoimmune disease, chronic liver disease, chronic kidney disease, hematologic disorder, alcohol use, or chronic medication exposure. In September 2025, chest CT incidentally revealed a right lower-lobe lung mass during evaluation for urinary retention. PET/CT on November 20, 2025 showed a hypermetabolic right lower-lobe lesion, mediastinal and bilateral hilar lymphadenopathy, and suspected right fourth and fifth rib metastases. CT-guided biopsy confirmed lung adenocarcinoma. PD-L1 tumor proportion score was approximately 1% by Ventana SP263 testing, and next-generation sequencing identified complex KRAS exon 2 mutations. Because the fourth and fifth rib lesions represented multiple extrathoracic metastases, the clinical stage was revised to cT1bN2M1c, stage IVB.

First-line therapy consisted of ivonescimab 1000 mg with nab-paclitaxel and cisplatin. Cycle 1 began on December 24, 2025, with ivonescimab on day 4. Cycles 2 and 3 included ivonescimab on day 2; the final ivonescimab dose was administered on February 11, 2026. Restaging before cycle 4 showed partial response, and the multidisciplinary team omitted ivonescimab from cycle 4 on March 4, 2026, giving chemotherapy alone.

### 2.2 Initial presentation and physical examination

On March 14, 2026, 10 days after the final chemotherapy and 31 days after the final ivonescimab dose, the patient developed fever to 39.3 °C with headache, sore throat, and rhinorrhea. On March 18, creatinine was 94 µmol/L and liver tests were normal. By March 20, creatinine had increased to 175 µmol/L. On March 22, jaundice and dark urine appeared; ALT was 179 U/L, AST 237 U/L, ALP 419 U/L, total bilirubin 19.5 µmol/L, albumin 27.9 g/L, and creatinine 375 µmol/L. Abdominal CT showed enlarged kidneys with perirenal exudation, mild biliary and pancreatic ductal dilatation, and gallbladder wall thickening. Urinalysis showed protein (++) and occult blood (+/-). On March 23, creatinine rose to 552-767 µmol/L, total bilirubin was 43.1 µmol/L, C-reactive protein was 20.7 mg/L, leukocytes were 2.17 ×10^9/L, hemoglobin was 106 g/L, and D-dimer was 874 ng/mL.

On admission, vital signs were temperature 36.3 °C, pulse 94 beats/min, respiratory rate 19 breaths/min, and blood pressure 122/75 mmHg. He was conscious and fluent but fatigued, with Glasgow Coma Scale score 14 and mild scleral icterus. No petechiae, ecchymoses, focal neurologic deficit, abdominal tenderness, rebound tenderness, or guarding was documented. Breath sounds were coarse bilaterally without obvious rales; cardiac auscultation did not reveal a significant murmur. Bilateral pitting edema was present in the lower extremities.

### 2.3 Diagnostic assessment

The initial differential diagnosis included severe infection, acute tubular injury, drug-induced liver injury, immune-related hepatitis/nephritis, biliary disease, chemotherapy-associated toxicity, and thrombotic microangiopathy (TMA). Meropenem, continuous renal replacement therapy (CRRT), and methylprednisolone 40 mg/day were started. MRCP showed cholecystitis, Glisson sheath edema, and ascites without obstructive jaundice. Autoimmune serology, hepatitis E IgM, hepatitis E RNA, and HBV DNA were negative or undetectable, and direct antiglobulin testing was negative. Despite improvement in inflammatory markers, cholestatic liver injury progressed, with total bilirubin reaching 293.2 µmol/L by March 31 and 440.4 µmol/L by April 13. Because liver and kidney biopsies were not performed, we describe the hepatic phenotype as severe cholestatic liver injury rather than biopsy-proven cholestatic hepatitis.

The hematologic evolution redirected the diagnosis. Peripheral smear on March 31 showed approximately 1% schistocytes. Plasma free hemoglobin was 50 mg/L on April 2. Progressive anemia, thrombocytopenia, schistocytes, Coombs-negative hemolysis, acute kidney injury, and elevated D-dimer raised concern for TMA. On April 14, rheumatology recommended ADAMTS13 activity, inhibitor testing, and complement assessment. On April 15, ADAMTS13 activity was 8.87% and functional inhibitor was 1.8 BU/mL, establishing acquired iTTP according to accepted diagnostic thresholds (5). Complement factor Ba was 1891 ng/mL, whereas soluble C5b-9 was 195 ng/mL, suggesting alternative pathway activation as a possible amplifier rather than proving primary complement-mediated atypical hemolytic uremic syndrome. Complement genetic testing was not performed; after severe ADAMTS13 deficiency with a functional inhibitor was confirmed, aHUS was considered less likely, although incomplete complement evaluation remains a limitation.

The PLASMIC score was calculated as 5 points: platelet count <30 ×10^9/L (+1), hemolysis evidence (+1), no active cancer (+0), no transplant (+1), MCV <90 fL (+0), INR <1.5 (+1), and creatinine <2.0 mg/dL at the time of iTTP work-up (+1). This intermediate score strengthened the need for urgent ADAMTS13 testing rather than excluding iTTP. The diagnostic evidence supporting iTTP and the PLASMIC score components are summarized in **Table 1**.

**Table 1:** Diagnostic evidence supporting iTTP and PLASMIC score breakdown.

Domain	Variable	Patient data	Interpretation
MAHA/TMA	Hemoglobin/platelets	Hb declined from 106 g/L to 57–68 g/L; platelets declined to 26 ×10^9/L	Anemia plus thrombocytopenia supported TMA rather than isolated cholestasis.
MAHA/TMA	Schistocytes	~1% on peripheral smear (Mar 31)	Compatible with microangiopathic hemolysis.
Hemolysis	LDH/reticulocytes/free hemoglobin	LDH 320 U/L; reticulocytes 1.11%; plasma free Hb 50 mg/L	Free Hb supported intravascular hemolysis; serial LDH and haptoglobin were not available.
Hemolysis	Bilirubin fraction	Peak TBiL 440.4 µmol/L; DBiL 346.9 µmol/L; indirect bilirubin ~93.5 µmol/L	Severe direct-predominant cholestasis with concurrent indirect component.
Immune hemolysis exclusion	Direct antiglobulin test	Negative	Argued against autoimmune hemolytic anemia as primary driver.
Coagulation	PT/INR, fibrinogen, D-dimer	PT 10.5 s, INR 0.94, fibrinogen 188 mg/dL, D-dimer 825 ng/mL; APTT not available	No overt disseminated intravascular coagulation pattern in available data.
Renal/urine	Creatinine and urinalysis	Creatinine peak 767 µmol/L; urinalysis protein (++), occult blood (+/-), WBC 30/µL, RBC 7/µL	KDIGO stage 3 AKI requiring temporary CRRT; renal function later normalized.
ADAMTS13	Activity/inhibitor	Activity 8.87%; inhibitor 1.8 BU/mL; repeat activity 23.82%, inhibitor 0.85 BU/mL on Apr 24	Confirmed acquired immune-mediated TTP; only one follow-up ADAMTS13 assessment was available.
Complement	Factor Ba/sC5b-9	Ba 1891 ng/mL; sC5b-9–195 ng/mL; complement genetic testing not performed	Suggested alternative pathway activation without proving primary complement-mediated TMA/aHUS.
PLASMIC	Platelet count <30 ×10^9/L	Yes, nadir 26 ×10^9/L	+1
PLASMIC	Hemolysis variable	Yes, indirect bilirubin >2 mg/dL equivalent and MAHA features	+1
PLASMIC	No active cancer	No; active stage IVB lung adenocarcinoma	+0
PLASMIC	No transplant	No solid-organ or stem-cell transplant	+1
PLASMIC	MCV <90 fL	No; MCV 93.6 fL before TTP diagnosis and 92.2 fL after diagnosis	+0
PLASMIC	INR <1.5	Yes; INR 0.94	+1
PLASMIC	Creatinine <2.0 mg/dL at iTTP work-up	Yes; creatinine 130 µmol/L on Apr 13 and 74 µmol/L on Apr 18	+1
PLASMIC	Total score	5	Intermediate clinical probability; ADAMTS13 testing was essential and diagnostic.

**Figure 1:**
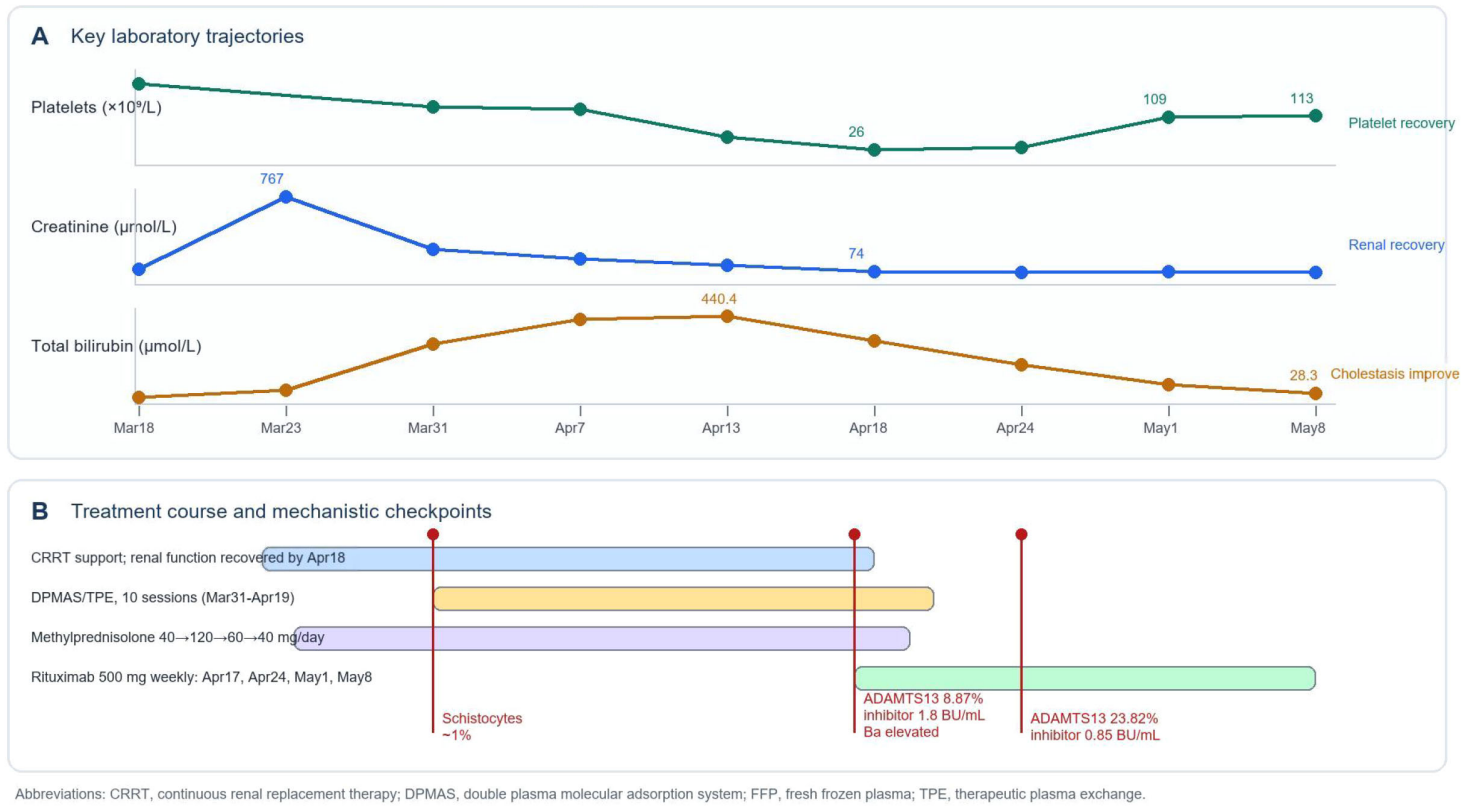
Integrated diagnostic and treatment timeline. **(A)** Dynamic trends in platelet count, serum creatinine, and total bilirubin from March 18 to May 8, 2026. The figure shows severe AKI with subsequent renal recovery, peak cholestatic liver injury, delayed platelet nadir, and later hematologic improvement. **(B)** Treatment course and mechanistic checkpoints, including CRRT support, DPMAS/TPE sessions, corticosteroid dosing, rituximab administration, first schistocyte detection, confirmatory ADAMTS13 activity/inhibitor testing, elevated factor Ba, and the available follow-up ADAMTS13 result. Abbreviations: CRRT, continuous renal replacement therapy; DPMAS, double plasma molecular adsorption system; FFP, fresh frozen plasma; TPE, therapeutic plasma exchange.

The renal course is important for accurate case framing. The patient had KDIGO stage 3 AKI requiring temporary CRRT, followed by recovery rather than dialysis dependence. Creatinine decreased to 276 µmol/L on March 31, 186 µmol/L on April 7, 130 µmol/L on April 13, and 74 µmol/L on April 18, with eGFR 92.7 mL/min/1.73 m². Thus, the kidney injury should be described as severe but reversible AKI requiring short-term CRRT support. The integrated diagnostic and treatment timeline is shown in **Figure 1**.

### 2.4 Therapeutic intervention and follow-up

Management evolved with the diagnostic formulation. Initial treatment emphasized infection control, renal support, hepatoprotection, and corticosteroids. On March 30-31, ivonescimab-associated immune-related liver and kidney injury was considered, immune therapy was permanently discontinued, methylprednisolone was escalated, and double plasma molecular adsorption system (DPMAS) artificial liver support was started. DPMAS reduced bilirubin burden but did not directly target the ADAMTS13 inhibitor.

Corticosteroid treatment was administered as follows: methylprednisolone 40 mg/day from March 25 to March 31, 120 mg/day from March 31 to April 13, 60 mg/day from April 14 to April 16, and 40 mg/day from April 17 to April 21. After iTTP was confirmed, treatment shifted toward mechanism-directed therapy. DPMAS/TPE support was performed for a total of 10 sessions between March 31 and April 19, 2026; after the ADAMTS13 result, the exchange strategy was redirected toward fresh frozen plasma (FFP)-based therapeutic plasma exchange to remove inhibitor and replenish ADAMTS13. The course therefore was not a fully standardized daily TPE protocol from the first TMA clue; rather, it reflects diagnostic evolution from cholestatic liver failure support to iTTP-directed plasma exchange. DPMAS/TPE was discontinued on April 19 after renal recovery, falling bilirubin, absence of new ischemic organ injury, and improving ADAMTS13/inhibitor results; formal clinical-response criteria could not be fully assessed because platelet normalization and serial LDH were unavailable.

Rituximab was administered as a fixed 500 mg intravenous dose on April 17, April 24, May 1, and May 8, 2026. The fixed-dose regimen reflected local clinical practice and concern for infection risk in a patient with advanced malignancy and recent severe systemic illness; it should not be interpreted as replacing the conventional body-surface-area-based rituximab regimen in guideline algorithms. Caplacizumab was not used because it was unavailable at the treating institution. No platelet transfusions were administered during the course because there was no life-threatening bleeding; red blood cell and plasma transfusions were given as clinically indicated. The treatment course and key laboratory milestones are summarized in **Table 2**.

**Table 2:** Treatment and laboratory timeline.

Date	Clinical/laboratory status	Treatment	Interpretation
Mar 14-23	Fever, respiratory symptoms, jaundice, dark urine, creatinine peak 767 µmol/L	Initial outpatient and emergency evaluation; broad differential including infection, drug injury, irAE, biliary disease, AKI	No TTP diagnosis yet
Mar 24-30	Admission with AKI, cholestatic liver injury, anemia, thrombocytopenia	Meropenem, CRRT, methylprednisolone 40 mg/day, hepatoprotective therapy	Renal support started
Mar 31-Apr 13	TBiL rose to 293.2 then 440.4 µmol/L; schistocytes ~1%; free Hb 50 mg/L	Methylprednisolone 120 mg/day; DPMAS support; IVIG Apr 5–9 per clinical record	TMA clues emerged but cholestatic/renal phenotype dominated
Apr 14-15	Rheumatology evaluation; ADAMTS13 8.87%, inhibitor 1.8 BU/mL; factor Ba 1891 ng/mL	Diagnosis revised to acquired iTTP with complement activation as a possible amplifier	Mechanism-directed treatment planned
Apr 17-Apr 19	Platelets reached nadir 26 ×10^9/L; creatinine normalized to 74 µmol/L; TBiL 307.3 µmol/L	FFP-based TPE emphasized; rituximab 500 mg Apr 17; steroids 40 mg/day; DPMAS/TPE course completed by Apr 19	Renal recovery; iTTP still hematologically active
Apr 24-May 8	ADAMTS13 23.82%, inhibitor 0.85 BU/mL on Apr 24; platelets 109-113 ×10^9/L by May 1-8; TBiL 28.3 µmol/L by May 8	Rituximab 500 mg weekly on Apr 24, May 1, May 8; no platelet transfusion	Laboratory improvement and partial hematologic recovery; formal clinical response not claimed

The available data support laboratory improvement and partial hematologic recovery, including improved ADAMTS13 activity, declining inhibitor titer, bilirubin reduction, sustained renal recovery, and platelet increase to 109-113 ×10^9/L. Formal clinical response was not claimed because platelet normalization and serial LDH <1.5 × upper limit of normal were not documented in the available case file (7).

## 3 Focused literature review and discussion

### 3.1 Literature search and screening

To make the review reproducible, PubMed was searched on July 8, 2026 using the exact string: ((“immune checkpoint inhibitor”[Title/Abstract] OR pembrolizumab[Title/Abstract] OR nivolumab[Title/Abstract] OR ipilimumab[Title/Abstract] OR atezolizumab[Title/Abstract] OR durvalumab[Title/Abstract] OR cemiplimab[Title/Abstract] OR avelumab[Title/Abstract]) AND (“thrombotic thrombocytopenic purpura”[Title/Abstract])). No language, date, or article-type limits were applied at the search stage. The search returned 22 records. Titles and abstracts were screened for ICI-associated TTP/iTTP; records were excluded if they were errata, non-TTP purpura/cytokine-release presentations, pharmacovigilance-only reports without individual patient data, or reports without sufficient clinical detail for comparison. Pharmacovigilance evidence was summarized separately from individual clinical reports. **Table 3** distinguishes confirmed iTTP from probable/broader TTP/TMA when ADAMTS13 details were incomplete. This focused review contextualizes the present case and should be interpreted alongside prior systematic or narrative reviews of ICI-associated TTP/TMA.

**Table 3:** Focused literature review of ICI-associated TTP/iTTP and comparison with the present case.

Study	Cancer/context	ICI/regimen	Timing/context	TTP/iTTP evidence	Treatment	Relevance
Moore 2022 (12)	FAERS pharmacovigilance	Multiple ICIs	Not patient-level	35 TTP reports; signals for atezolizumab, nivolumab, pembrolizumab	Not applicable	Supports class-level safety signal
King 2017 (13)	Melanoma	Ipilimumab	Reported after CTLA-4 blockade	TTP; ADAMTS13 details limited in abstract	TPE, steroids; outcome reported in case	Early proof-of-concept for ICI-associated TTP
Youssef 2018 (14)	Solid tumor context	Checkpoint inhibitors	After ICI exposure	TTP attributed to checkpoint inhibitors	TPE/steroids used in reported case	Broadened recognition across ICIs
Gergi 2020 (15)	Anal squamous cell carcinoma	Nivolumab	After PD-1 blockade	iTTP phenotype	TPE/immunosuppression	Shows PD-1-associated hematologic toxicity
Lancelot 2021 (16)	Cancer treated with ICI	Checkpoint inhibitor immunotherapy	After ICI exposure	Refractory TTP	TPE and intensified immunosuppression	Highlights potential severity and treatment resistance
De Filippis 2021 (17)	Metastatic NSCLC	ICI therapy	Lung cancer context	ICI-associated TTP	TPE, steroids, supportive care	Clinically close oncologic setting
Nelson 2022 (18)	Cancer treated with pembrolizumab	Pembrolizumab	During pembrolizumab therapy	TTP associated with pembrolizumab	TPE/immunosuppression	Supports pembrolizumab class comparison
Mullally 2022 (19)	Metastatic melanoma	Ipilimumab + nivolumab	After dual checkpoint blockade	TTP reported with severe course	TPE and immunosuppression	Demonstrates fatal potential and diagnostic urgency
Kozak 2023 (20)	Cancer treated with pembrolizumab	Pembrolizumab	During pembrolizumab therapy	TTP with ADAMTS13 monitoring	Extended TPE + rituximab	Durable remission supports ADAMTS13-guided follow-up
Yoshida 2024 (21)	Gastric tube cancer	Nivolumab	After nivolumab	Nivolumab-induced TTP	TPE/immunosuppression	Further PD-1 case evidence
Palega 2026 (22)	Pembrolizumab-treated cancer	Pembrolizumab	After PD-1 exposure	Case report and literature review	TPE/immunosuppression per case	Recent synthesis; useful comparator
Ke 2026 (23)	Triple-negative breast cancer	PD-L1 inhibition + taxane	Chemoimmunotherapy context	TTP during PD-L1/taxane therapy	TPE/steroids/rituximab reported	Most analogous combination-therapy attribution challenge
Present case	Stage IVB lung adenocarcinoma	Ivonescimab + nab-paclitaxel/cisplatin	35 days after final ivonescimab dose; after chemotherapy-only cycle	ADAMTS13 8.87%, inhibitor 1.8 BU/mL; factor Ba high	DPMAS/TPE 10 sessions, FFP-based exchange after diagnosis, steroids, rituximab; caplacizumab unavailable	Suggests iTTP can emerge in a PD-1/VEGF bispecific setting with severe cholestatic injury and reversible AKI

### 3.2 Interpretation of the present case

The present case does not prove that ivonescimab alone caused iTTP. The patient also received cisplatin and nab-paclitaxel, had fever and possible infection, and did not undergo liver or kidney biopsy. We therefore frame the event as iTTP after ivonescimab-containing chemoimmunotherapy. Nevertheless, the timing after PD-1/VEGF exposure, severe ADAMTS13 activity deficiency below 10%, positive functional inhibitor, Coombs-negative hemolysis, schistocytes, platelet nadir of 26 ×10^9/L, and improvement after plasma exchange plus rituximab support an acquired immune-mediated iTTP phenotype rather than isolated cisplatin nephrotoxicity, chemotherapy-associated myelosuppression, or nonspecific critical illness.

A plausible model involves converging mechanisms, but it remains speculative. PD-1 blockade may reduce peripheral tolerance and promote T-cell and B-cell autoimmunity, enabling anti-ADAMTS13 autoantibody formation. VEGF inhibition may injure glomerular endothelial homeostasis and sensitize the renal microvasculature to thrombotic microangiopathy (24, 25). Elevated factor Ba suggests alternative complement pathway activation, which may have amplified endothelial injury. However, this mechanistic interpretation is hypothesis-generating only because liver and kidney tissue were unavailable, complement markers were not serially profiled, and complement genetic testing was not performed.

The differential diagnosis also included chemotherapy-associated or drug-induced TMA. Drug-induced TMA is mechanistically heterogeneous and may reflect immune-mediated reactions or direct endothelial toxicity (26, 27). However, profound ADAMTS13 deficiency with a functional inhibitor is much more supportive of acquired iTTP than of typical platinum-associated endothelial injury. This distinction matters because organ support, DPMAS, or glucocorticoids alone cannot replace ADAMTS13 or remove circulating inhibitors.

### 3.3 Clinical lessons

• In patients receiving ICIs or PD-1/VEGF-directed therapy, new thrombocytopenia plus anemia should trigger peripheral smear review, hemolysis testing, PLASMIC scoring, and early ADAMTS13 testing rather than attribution only to chemotherapy, infection, or liver dysfunction.
• A PLASMIC score may be intermediate in patients with active malignancy or MCV >90 fL; an intermediate score should not stop ADAMTS13 testing when MAHA and organ injury are present.
• Severe AKI requiring CRRT should be distinguished from dialysis-dependent kidney failure. In this patient, kidney function normalized rapidly after temporary support and systemic control.
• For confirmed iTTP, FFP-based TPE and immunosuppression are mechanism-directed therapies; ADAMTS13 recovery and inhibitor decline should be interpreted alongside platelet count and LDH.

## 4 Limitations

This report has limitations. Liver and kidney biopsies were not obtained because of thrombocytopenia and clinical instability, limiting tissue-level attribution among immune-related liver injury, acute tubular injury, renal TMA, and anti-VEGF endothelial toxicity. This is particularly important because anti-VEGF therapy can independently cause renal endothelial injury and TMA. Haptoglobin, APTT, and serial LDH data were not available in the extracted records, and platelet normalization was not documented within the presented follow-up window. ADAMTS13 activity and inhibitor testing were repeated once, but serial ADAMTS13 monitoring beyond April 24 was not available; complement markers were not measured serially, and complement genetic testing was not performed. The patient received combination therapy and had infection-like symptoms, making it impossible to isolate ivonescimab from chemotherapy or inflammatory triggers. Finally, the literature review is focused and clinically contextual rather than a systematic review, and longer follow-up is needed to determine iTTP relapse risk, long-term kidney outcome, and subsequent oncologic treatment strategy.

## 5 Conclusion

We describe ADAMTS13 inhibitor-positive acquired iTTP with severe cholestatic liver injury and reversible AKI after ivonescimab-containing chemoimmunotherapy. Causality should be interpreted cautiously because the event occurred after a combination regimen, and attribution to ivonescimab alone cannot be established without tissue confirmation, serial complement profiling, or re-exposure. The renal event should be framed as severe AKI requiring temporary CRRT rather than persistent or dialysis-dependent renal failure. In the era of PD-1/VEGF bispecific antibody-containing regimens, clinicians should maintain a low threshold for smear review, hemolysis testing, PLASMIC scoring, and ADAMTS13 activity/inhibitor assays when thrombocytopenia, MAHA, and organ injury emerge together.

## Data Availability

The original contributions presented in the study are included in the article/**Supplementary Material**. Further inquiries can be directed to the corresponding authors.

## Supplementary material

Supplementary Figure 1 Dynamic changes in scleral icterus. Scleral/conjunctival icterus during peak cholestatic liver injury and subsequent improvement. Written informed consent for publication of clinical images was obtained.
